# Efficacy and safety of different chemotherapy regimens concurrent with radiotherapy in the treatment of locally advanced cervical cancer

**DOI:** 10.1186/s12885-024-12358-8

**Published:** 2024-05-15

**Authors:** Yaping Wu, Peng Jiang, Zhiying Chen, Wei Li, Bin Dong, Yongchun Zhang

**Affiliations:** Department of Radiation Oncology, The Affiliated Hospital of Qingdao University, 16 Jiangsu Road, Qingdao, 266003 China

**Keywords:** Nab-paclitaxel, Locally advanced cervical cancer (LACC), Concurrent chemoradiotherapy (CCRT), Efficacy, Adverse events (AEs)

## Abstract

**Background:**

Evaluate the efficacy and safety of different chemotherapy regimens concurrent with radiotherapy in treating locally advanced cervical cancer (LACC).

**Methods:**

Retrospective data was collected from LACC patients who were treated at our institution. These patients were categorized into three groups: the single-agent cisplatin (DDP) chemoradiotherapy group, the paclitaxel plus cisplatin (TP) chemoradiotherapy group, and the nanoparticle albumin-bound (nab-) paclitaxel combined with cisplatin (nPP) chemoradiotherapy group. The primary endpoints were overall survival (OS) and progression-free survival (PFS) and the secondary endpoints were objective response rate (ORR) and incidence of adverse events (AEs).

**Results:**

A total of 124 patients were enrolled (32 in the DDP group, 41 in the TP group, and 51 in the nPP group). There were differences in OS (*P* = 0.041, HR 0.527, 95% CI 0.314–0.884) and PFS (*P* = 0.003, HR 0.517, 95% CI 0.343–0.779) between the three groups. Notably, the 2-year OS rate was significantly higher in the nPP group compared to the DDP group (92.2% vs. 85.4%, *P* = 0.012). The 2-year PFS rates showed a marked increase in the TP group (78.0% vs. 59.4%, *P* = 0.048) and the nPP group (88.2% vs. 59.4%, *P* = 0.001) relative to the DPP group, with multiple comparisons indicating that the 2-year PFS rate was significantly superior in the nPP group versus the DDP group (88.2% vs. 59.4%, *P* = 0.001). Moreover, the ORR was also significantly higher in the nPP group than in the DDP group (*P* = 0.013); and no statistically significant differences were found in the incidence of AEs among the groups (*P* > 0.05).

**Conclusions:**

In LACC treatment, the two cisplatin-based doublet chemotherapy regimens are associated with better outcomes, with the nab-paclitaxel plus cisplatin regimen showing better efficacy than the paclitaxel plus cisplatin regimen. Furthermore, the AEs associated with these regimens were deemed tolerable. These findings could provide a reference for the clinical treatment of LACC. However, further prospective studies are needed to verify it.

## Background

Cervical cancer is the fourth most frequently diagnosed cancer and the fourth leading cause of cancer death in women, representing a major global health challenge. According to statistics, there are about 600,000 new cases of cervical cancer worldwide every year, and nearly 90% of them occur in low-income and middle-income countries [1, 2]. Although early-stage cervical cancer is often curable, 40-50% of patients are diagnosed at the locally advanced stage [International Federation of Obstetricians and Gynecologists (FIGO2009/2018), IB2-IVA stage/IB3-IVA stage] [3, 4]. Cisplatin-based concurrent chemoradiotherapy combined with brachytherapy is the standard treatment for LACC. After completion of CCRT, the 5-year OS of patients is about 65–70%, and nearly 40% have recurrence or metastasis [5, 6]. Reducing distant metastasis and improving the long-term survival of LACC patients is still a clinical challenge. Many studies have been conducted at home and abroad to improve the efficacy of concurrent chemotherapy regimens in treating LACC, but which regimen is better is not conclusive. In previous studies, paclitaxel combined with platinum has shown potent activity and is a commonly used combination chemotherapy regimen; however, the increased incidence of adverse reactions reduced patient compliance [7–9]. Nab-paclitaxel is an albumin-bound, solvent-free form of paclitaxel in nanoparticles, which is now widely used for the treatment of breast cancer, locally advanced or metastatic non-small cell lung cancer, and ovarian cancer [10]. Moreover, the efficacy in drug-resistant, metastatic, and recurrent cervical cancer has also been demonstrated [11]. However, there are few reports on its use in treating LACC. In this study, nab-paclitaxel was used in CCRT for LACC and investigated the efficacy and safety of different chemotherapy regimens concurrent radiotherapy for LACC.

## Methods

### Patient characteristics

The clinical data of 124 patients with LACC who underwent CCRT and intracavitary brachytherapy (ICBT) in our hospital from March 2018 to January 2021 were retrospectively analyzed. The patients were divided into three groups according to different treatment protocols: 32 patients in the DDP group, 41 in the TP group and 51 in the nPP group.

The inclusion criteria were as follows: (I) Diagnosed with cervical cancer through histopathological examination, including cervical squamous cell carcinoma and cervical adenocarcinoma; (II) stage IB3–IVA; (III) no previous surgical treatment; (IV) no previous history of radiotherapy; (V) no bone marrow suppression, and liver and kidney functions were generally normal.

The exclusion criteria were: (I) Presence of other cancer types; (II) pregnant or lactating women; (III) incomplete data on clinical treatment; (IV) severe complications or severe infection in important organs such as heart and lung; (V) change of chemotherapy regimen during the course of treatment.

Gynecological examination, magnetic resonance imaging (MRI), positron emission computed tomography (PET-CT), computed tomography (CT), or Ultrasound were used to evaluate the local tumor, lymph node status, and tumor metastasis. The maximum diameter of a tumor is measured based on CT or MRI scan results prior to treatment. There was no statistical difference in baseline data such as age, mass size, pathological type, clinical stage, and initial hemoglobin among the three groups (*P* > 0.05). (Table 1)

**Table 1:** Characteristics of the 124 patients enrolled in the study (n/%)

	DDP Group (*N* = 32)	TP Group (*N* = 41)	nPP Group (*N* = 51)	*P* value
Age mean ± SD (years)	56.63 ± 9.75	54.37 ± 9.26	52.86 ± 10.80	0.256
Tumor size				
≤ 4 cm	14 (43.8%)	13 (31.7%)	19 (37.3%)	0.572
>4 cm	18 (56.3%)	28 (68.3%)	32 (62.7%)	
Histology				
Squamous cell carcinoma Adenocarcinoma	28(87.5%) 4 (12.5%)	38 (92.7%) 3(7.3%)	48 (94.1%) 3 (5.9%)	0.643
Stage (FIGO 2018)				
IB3 IIA2 IIB IIIA IIIB	4 (12.5%) 4 (12.5%) 8 (25%) 1 (3.1%) 5 (15.6%)	4 (9.8%) 3 (7.3%) 11 (26.8%) 2 (4.9%) 5 (12.2%)	6 (11.8%) 6 (11.8%) 9 (17.6%) 2 (3.9%) 3 (5.9%)	0.538
IIIC	8 (25%)	14 (34.1%)	19 (37.3%)	
IVA	2 (6.3%)	2 (4.9%)	6 (11.8%)	
Hemoglobin (g/L)				
80–110 ≥ 110	10 (31.3%) 22 (68.8%)	10 (24.4%) 31 (75.6%)	15 (29.4%) 36 (70.6%)	0.788

SD, standard deviation; FIGO, International Federation of Gynecologists and Obstetricians.

### Treatment

Following the careful exclusion of contraindications to chemoradiotherapy, patients proceeded with radiation therapy localization and treatment planning, concurrently initiating a cycle of systemic chemotherapy. In the DDP group, patients were administered a single dose of 75 mg/m² of single-agent cisplatin, repeated on a three-week interval. For the TP group, patients received a combination of paclitaxel and cisplatin, with a single dose consisting of 135 mg/m² of paclitaxel and 75 mg/m² of cisplatin, also repeated at three-week intervals. As for the nPP group, patients were treated with a combination of nab-paclitaxel and cisplatin, receiving a single dose of 200 mg/m² of nab-paclitaxel and 75 mg/m² of cisplatin, again on a three-weekly repetition schedule. Throughout the radiotherapy phase, chemotherapy was synchronously administered once every three weeks, approximating to two complete cycles of concurrent chemotherapy. Within one week following radiotherapy, an additional 0 to 1 cycle of chemotherapy was given as needed, culminating in an overall total of 3 to 4 chemotherapy cycles for each patient.

**Fig. 1:**
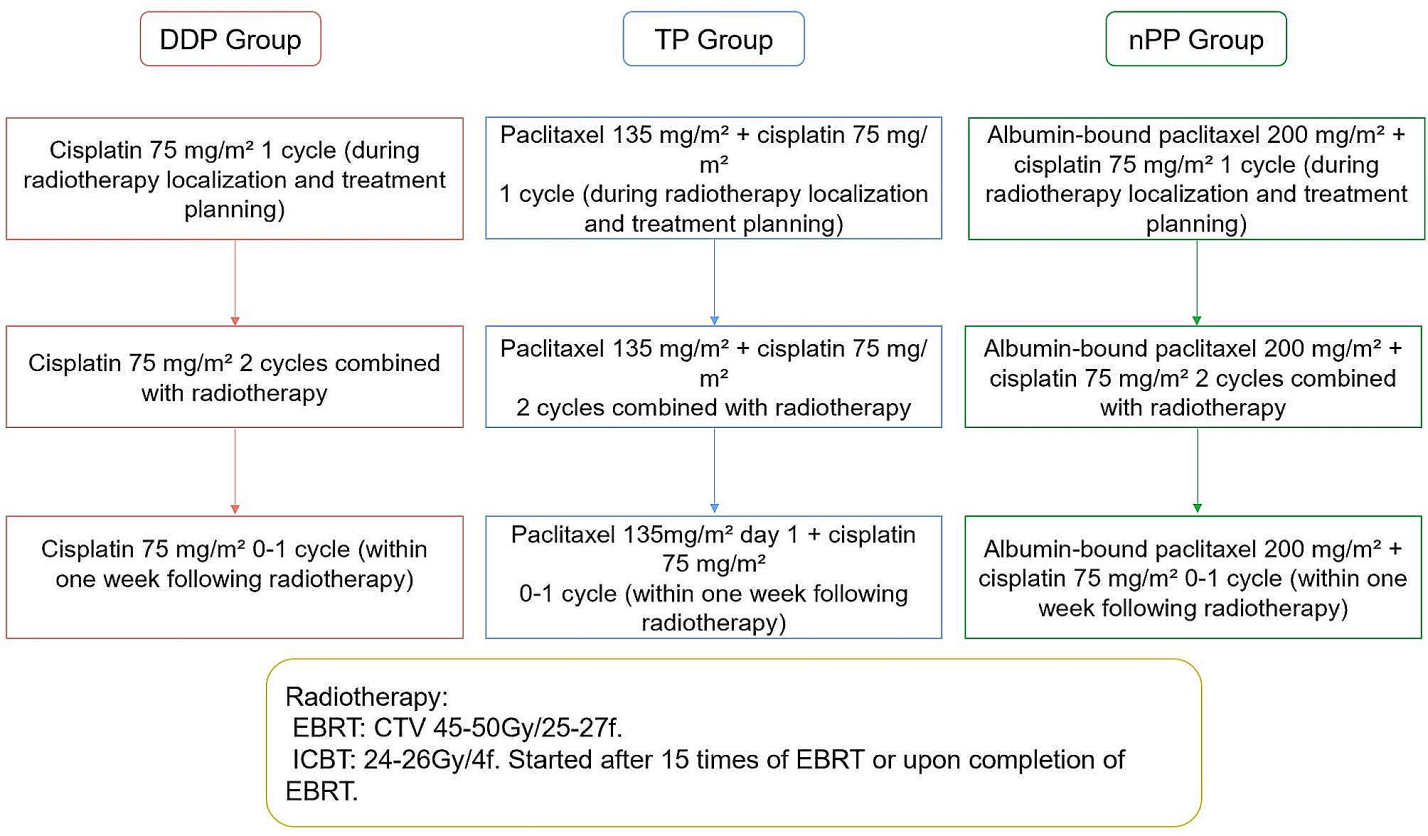
Treatment flow diagram. CCRT, concurrent chemoradiotherapy; EBRT, external beam radiation therapy; CTV, clinical target volume; CTV-n, gross target volume of lymph nodes; ICBT, intracavitary brachytherapy

Radiotherapy (RT) included external beam radiation therapy (EBRT) and intracavitary brachytherapy. In EBRT, Image-guided Intensity-Modulated Radiation Therapy was utilized. The patient was positioned in the supine posture secured with thermoplastic immobilization molds. Enhanced CT scans were performed at a slice thickness of 5 millimeters, with the superior boundary at the level of the upper margin of the tenth thoracic vertebra and the inferior boundary approximately 10 centimeters below the ischial tuberosity. The clinical target volume (CTV) included the primary cervical lesion area and the lymph node area, encompassing the entire cervix, parametria, uterine corpus, partial or complete vagina, as well as the draining lymph nodes of the obturator, internal iliac, external iliac, common iliac, and presacral regions. Depending on the specific case, the inguinal and para-aortic lymph node drainage areas may or may not be included. The planning target volume (PTV) was defined as an expansion of 3–5 millimeters from the CTV to establish the planning treatment volume. The dose of CTV was 45.0 ~ 50 Gy/25-27fraction (f), for patients with positive parametrial involvement, a simultaneous integrated boost up to 58–62 Gy/25-27f was administered locally. Once the primary tumor shrinks to less than 3 cm in diameter, typically after about 15 fractions of EBRT or upon completion of EBRT, three-dimensional image-guided high-dose-rate brachytherapy was conducted under CT guidance, with a dose of 24–26 Gy/4f. The equivalent dose of 2 Gy (EQD2) of ERBT combined with ICBT was 82–88 Gy. The total treatment course spanned approximately 7 weeks. The treatment flow diagram of the three groups is shown in Fig. 1.

Chemotherapy dose was maintained or reduced after giving symptomatic treatment to patients who developed hepatic dysfunction, severe gastrointestinal reactions, or bone marrow suppression after treatment. If the patient could not tolerate the adverse effects of the treatment, the chemotherapy was stopped.

### Evaluation of tumor response

Short-term efficacy: Efficacy was evaluated according to the Response Evaluation Criteria in Solid Tumors (RECIST 1.1). The short-term outcomes were classified into complete response (CR), partial response (PR), stable disease (SD), progressive disease (PD), and objective response rate (ORR) = (CR + PR)/total × 100%.

Long-term efficacy: Patients were subjected to routine medical reviews and telephonic check-ups every 3–6 months post-treatment. All subjects received a minimum of two years’ follow-up, or until their demise, whichever occurred earlier. Overall Survival (OS) was defined as the duration from the initiation of treatment until death due to any cause or until the designated endpoint of follow-up. Progressive Free Survival (PFS) was determined as the interval between the start of treatment and the first instance of disease progression, death from any cause, or the end of the follow-up period.

AEs: AEs were assessed according to the Common Terminology Criteria for Adverse Events version 5.0 (CTCAE v5.0), mainly including bone marrow suppression, gastrointestinal reactions, allergic reaction, peripheral neurotoxicity, radiation enteritis, radiation cystitis and hepatic impairment and renal impairment. with a focus on key manifestations such as bone marrow suppression, gastrointestinal adverse reactions, allergic responses, peripheral neuropathy, radiation-induced enteritis, radiation cystitis, as well as hepatic and renal dysfunctions.

### Statistical analysis

SPSS 26.0 (IBM, USA) was used for the statistical analysis. Measured data were expressed as mean ± standard deviation. One-way analysis of variance (ANOVA) was used for comparisons between groups. The quantitative index was converted into frequency and percentage. The chi-square test was used for comparisons among the three groups. OS and PFS were estimated using the Kaplan–Meier method, and the Log-rank test compared differences in survival curves. *P* < 0.05 (two-tailed) was considered significant. Using Bonferroni adjustment method (test level a = 0.05/3 = 0.017, *P*<0.017) made multiple comparisons between the three groups.

## Results

All patients completed EBRT and ICRT, and two patients in the TP group discontinued chemotherapy after one cycle due to adverse reactions. The differences in the number of chemotherapy cycles and EBRT, ICRT, and EQD2 doses were not statistically significant among the three groups. (Table 2)

**Table 2:** Treatment comparisons for the three groups

	DDP Group	TP Group	nPP Group	*P* value
Cycles of chemotherapy	3.28 ± 0.81	3.41 ± 0.77	3.73 ± 1.02	0.067
EBRT				
Dose of CTV, Gy	52.72 ± 4.72	54.07 ± 5.31	54.71 ± 5.21	0.218
ICBT				
EQD2, Gy	83.13 ± 1.83	83.34 ± 1.98	83.22 ± 1.97	0.891
Radiotherapy, days	47.19 ± 4.15	48.02 ± 4.59	47.41 ± 4.16	0.679

SD, standard deviation; EBRT, external beam radiation therapy; CTV, clinical target volume; ICBT, intracavitary brachytherapy; EQD2, equivalent dose in 2 Gy/fraction.

### Short-term efficacy

In the DDP group, 17 cases (53.1%) achieved CR, 6 cases (18.8%) achieved PR, and ORR was 71.9% (23/32). In the TP group, 25 cases (61.0%) achieved CR, 10 cases (24.4%) achieved PR, and ORR was 85.4% (35/41). In the nPP group, 37 cases (72.5%) achieved CR, 10 cases (19.6%) achieved PR, and ORR was 92.2% (47/51). There was a difference in ORR between the three groups (71.9% vs. 85.4% vs. 92.2%, *P* = 0.044). Multiple comparisons revealed that the ORR was significantly higher in the nPP group than in the DDP group (71.9% vs. 92.2%, *P* = 0.013). In contrast, no statistically significant difference was seen in the TP group compared with the DDP group (85.4% vs. 71.9%, *P* = 0.157). (Table 3)

**Table 3:** Short-term efficacy (n/%)

	DDP Group (*N* = 32)	TP Group (*N* = 41)	nPP Group (*N* = 51)	*P* value		
				DDP vs. TP vs. nPP	TP vs. DDP	nPP vs. DDP
CR	17 (53.1%)	25 (61.0%)	37 (72.5%)	0.182	0.501	0.071
PR	6 (18.8%)	10 (24.4%)	10 (19.6%)	0.802	0.563	0.923
SD	4 (12.5%)	4 (9.8%)	1 (2.0%)	0.121	0.723	0.070
PD	5 (15.6%)	2 (4.9%)	3 (5.9%)	0.235	0.228	0.250
ORR	23 (71.9%)	35 (85.4%)	47(92.2%)	0.044	0.157	**0.013** ^*^

CR, complete response; PR, partial response; ORR, objective response rate (CR + PR); SD, stable disease; PD, progressive disease.

### Long-term efficacy

**Fig. 2:**
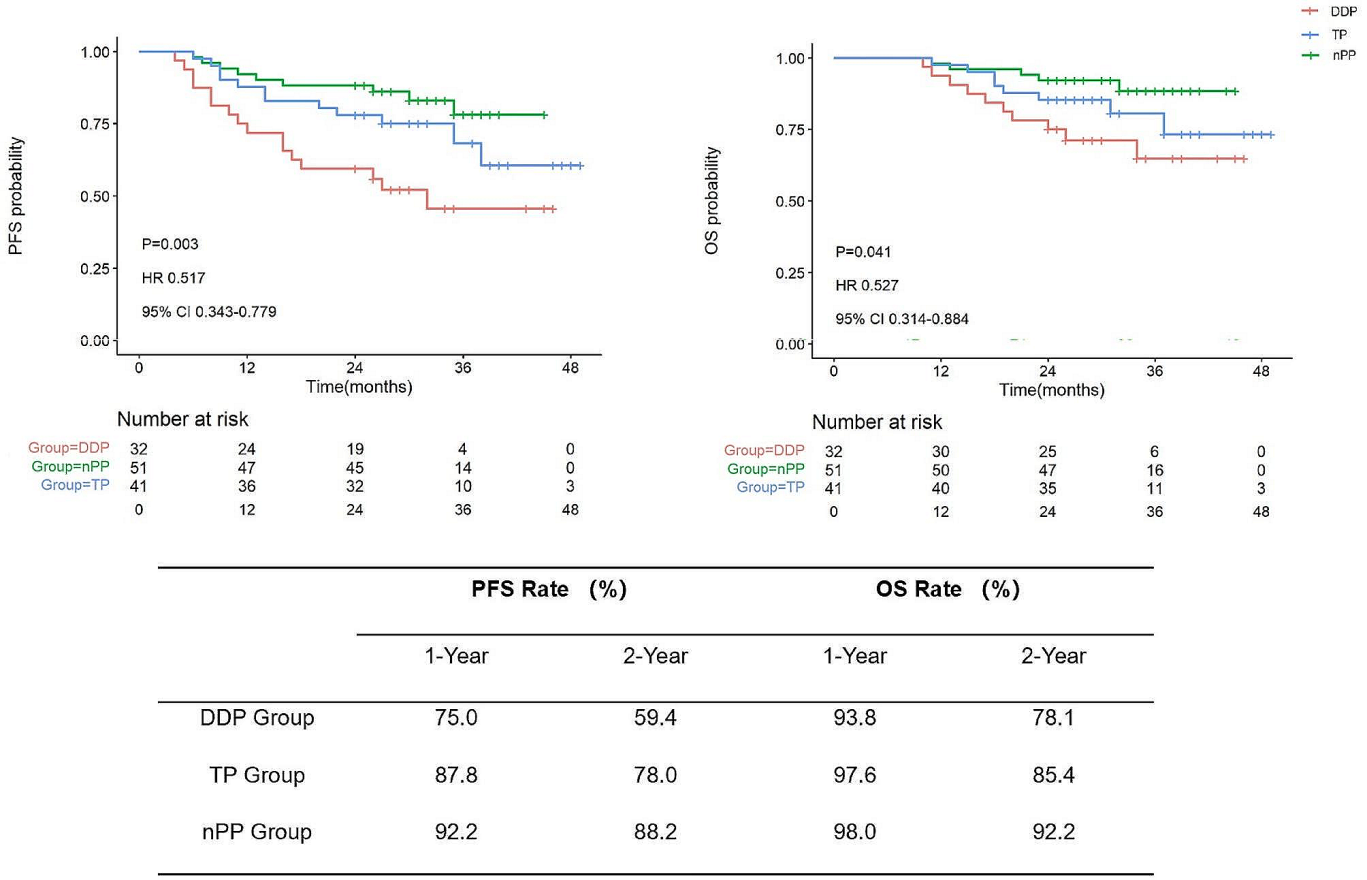
Kaplan-Meier analysis of the three groups, and percentages of 1-year and 2-year PFS and OS for patients in the three group. PFS, progression-free survival; OS, overall survival

Survival follow-up was until January 2023, with a median follow-up of 30 months (10–49 months). The 1- and 2-year OS rates were 93.8% vs. 97.6% vs. 98.0% and 78.1% vs. 85.4% vs. 92.2% in the DDP, TP, and nPP groups, respectively, with statistically significant differences (*P* = 0.041, log-rank test). 1- and 2-year PFS rates were 75.0% vs. 87.8% vs. 92.2% and 59.4% vs. 78.0% vs. 88.2% in the DDP, TP, and nPP groups, respectively, with statistically significant differences (*P* = 0.003, log-rank test). (Fig. 2)

The 2-year OS rate in the nPP group was significantly higher than in the DDP group (92.2% vs. 78.1%, *P* = 0.012, HR = 0.525, 95%CI = 0.307–0.899). There was no significant difference in the 2-year OS rate between the TP group and the DDP group (85.4% vs. 78.1%, *P* = 0.237). The 2-year PFS rates were higher in both the TP groups (78.0% vs. 59.4%, *P* = 0.048) and the nPP groups (88.2% vs. 59.4%, *P* = 0.001) than in the DDP group, but multiple comparisons suggested that the 2-year PFS rate was higher in the nPP group compared with the DDP group (88.2% vs. 59.4%, *P* = 0.001, HR = 0.525, 95% CI = 0.348–0.791). (Table 4)

**Table 4:** Two-Year PFS and OS among all included patients

	nPP Group vs.TP Group vs. DDP Group				TP Group vs. DDP Group				nPP Group vs. DDP Group		
	*P* value	HR	95%CI		*P* value	HR	95%CI		*P* value	HR	95%CI
2-Year-OS	**0.041**	**0.527**	**0.314–0.884**		0.237	0.575	0.227–1.459		**0.012**	**0.525**	**0.307–0.899**
2-Year-PFS	**0.003**	**0.517**	**0.343–0.779**		0.048	0.480	0.227–1.016		**0.001**	**0.525**	**0.348–0.791**

OS, overall survival; PFS, progression-free survival; HR: hazard ratio; CI: confidence interval.

### AEs

AEs were tolerable in all three groups, and the most common AEs were myelosuppression and gastrointestinal reactions. There was no significant difference in AEs between the groups (*P* > 0.05). (Table 5)

**Table 5:** Adverse events in the three groups

	DDP Group	TP Group	nPP Group	*P* value
Bone marrow suppression				0.555
Stage 0–2 Stage 3–4	18 (56.3%) 14 (43.8%)	22 (53.7%) 19 (46.3%)	23 (45.1%) 28 (54.9%)	
Gastrointestinal reactions				0.596
Stage 0–2 Stage 3–4	24 (75.0%) 8 (25.0%)	29 (70.7%) 12 (29.3%)	33 (64.7%) 18 (35.3%)	
Allergic reaction				0.134
No occurrence	30 (93.8%)	32 (78.0%)	45 (88.2%)	
Occurrence	2 (6.3%)	9 (22.0%)	6 (11.8%)	
Peripheral neurotoxicity				0.257
No occurrence	26 (81.3%)	30 (73.2%)	33 (64.7%)	
Occurrence	6 (18.8%)	11 (26.8%)	18 (35.3%)	
Radiation enteritis				0.985
Stage 0 Stage 1–2 Stage 3	14(43.8%) 16(50.0%) 2(6.3%)	17 (41.5%) 21 (51.2%) 3 (7.3%)	24 (47.1%) 23 (45.1%) 4 (7.8%)	
Radiation cystitis				0.844
No occurrence	25 (78.1%)	31 (75.6%)	37 (72.5%)	
Occurrence	7 (21.9%)	10 (24.4%)	14 (27.5%)	
Hepatic impairment				0.241
No occurrence	30 (93.8%)	34 (82.9%)	41 (80.4%)	
Occurrence	2 (6.3%)	7 (17.1%)	10 (19.6%)	
Renal impairment				0.405
No occurrence	31 (96.9%)	36 (87.8%)	46 (90.2%)	
Occurrence	1 (3.1%)	5 (12.2%)	5 (9.8%)	

## Discussion

National Comprehensive Cancer Network (NCCN) clinical practice guidelines for cervical cancer have recommended cisplatin-based CCRT as standard treatment for LACC [12] after five large-sample randomized controlled trials conducted by Gynecologic Oncology Group (GOG), Radiation Therapy Oncology Group (RTOG), and Southwest Oncology Group (SWOG) in the United States reported that concurrent radiotherapy could improve survival in cervical cancer [13]. However, many patients have residual tumors after treatment, leading to tumor progression or death.

To enhance the therapeutic effect and prognosis of cervical cancer, a variety of novel treatment strategies are actively being explored. The development of molecular targeted therapies has opened up new avenues. The GOG 240 Phase III clinical trial combined bevacizumab with chemotherapy for Stage IVB/recurrent/refractory cervical cancer patients, significantly improving their OS rate [14]. And based on this study, the Food and Drug Administration (FDA) approved bevacizumab for the first-line treatment of recalcitrant/recurrent/metastatic cervical cancer. In the RTOG 0417 phase II trial [15], bevacizumab combined with chemoradiotherapy was applied to stage IB to IIIB cervical cancer patients. The 3-year OS and disease-free survival (DFS) were 81.3% and 68.7%, respectively, while the incidence of grade 3 and 4 adverse events was 26.5% and 10.2%, respectively. However, since this treatment did not demonstrate superiority over historical controls with standard cisplatin chemoradiotherapy, further investigation with bevacizumab was not pursued. Bevacizumab increased the incidence of AEs such as hypertension, thrombosis, and gastrointestinal fistula while improving its efficacy [16], and its use has been greatly limited as drug resistance has developed. Immune checkpoint inhibitors are currently a hot spot of research in various oncology therapies. PD-1/PD-L1 inhibitors have shown remarkable efficacy in treating recurrent/metastatic cervical cancer [17, 18], and the FDA has approved pembrolizumab and nivolumab for treating recurrent/metastatic cervical cancer. Regarding immunotherapy for LACC, the CALLA Phase III clinical trial combined durvalumab or placebo with concurrent chemoradiotherapy in treating LACC patients [19], however, the results indicated that durvalumab combined with CCRT failed to improve the PFS of LACC patients. The KEYNOTE-A18 Phase III trial results suggested that combining immunotherapy with CCRT might achieve synergistic effects in patients with locally advanced cervical cancer. While targeted therapy and immunotherapy remain limited in their application for LACC, cytotoxic chemotherapy remains indispensable in the treatment of LACC [20]. 

Petrelli et al. [21] conducted a meta-analysis of 1500 patients, demonstrating that the combination of CCRT with a cisplatin-based dual agent significantly improved the OS (*P* = 0.0002, OR 0.65, 95%CI 0.51–0.81) and PFS (*P* = 0.006, OR 0.71, 95% CI 0.55–0.91) compared to weekly cisplatin single-agent CCRT. Similarly, another meta-analysis published by Ma et al. [22] showed that CCRT with a platinum-based doublet significantly improved OS (*P* = 0.01, HR 0.75, 95% CI 0.60–0.94) and PFS (*P* = 0.01, HR 0.78, 95% CI 0.65–0.94) compared with CCRT combined with cisplatin monotherapy. Consistent with past research, the combination of paclitaxel and platinum is often the favored chemotherapy regimen for treating LACC considering its impact on ORR and PFS [8]. However, the traditional formulation of paclitaxel is associated with reduced patient adherence due to its high frequency of AEs, such as myelosuppression and allergic reactions, thereby impacting overall treatment outcomes [23], and in this study, two patients in the TP group discontinued concurrent chemotherapy due to AEs of chemotherapy. Nab-paclitaxel, a 130 nano-meter albumin-bound paclitaxel complex, binds to specific receptors on the surface of tumor vascular endothelial cells, facilitating the uptake of paclitaxel into tumor cells via albumin-mediated endocytosis. It increases the concentration of paclitaxel in the tumor stroma, aggregates more anti-tumor drugs to the lesion, and ultimately enhances treatment outcomes. Furthermore, the absence of the requirement for co-solvents and desensitization pretreatment renders nab-paclitaxel more convenient and safer to administer [24]. 

Our study used nab-paclitaxel in CCRT to compare the efficacy and safety of chemotherapy regimens of single-agent cisplatin, paclitaxel combined with cisplatin, and nab-paclitaxel combined with cisplatin concurrent radiotherapy for LACC. The results showed differences in OS (*P* = 0.041, HR 0.527, 95%CI 0.314–0.884) and PFS (*P* = 0.003, HR 0.517, 95%CI 0.343–0.779) rates among the three groups. The 2-year OS rate was higher in patients in the nPP group than in the DDP group (92.2% vs. 78.1, *P* = 0.012 < 0.017, HR 0.525, 95% CI 0.307–0.899). However, there was no significant difference in the 2-year OS rate between the TP group and the DDP group (85.4 vs. 78.1%, *P* = 0.237), which may be related to the short follow-up time. An extended follow-up duration may be able to reveal a statistically discernible disparity in OS rates between the two groups. The 2-year PFS rate was higher in both the TP and nPP groups than in the DDP group (78.0% vs. 59.4%, *P* = 0.048; 88.2% vs. 59.4%, *P* = 0.001), and multiple comparisons suggested that the 2-year PFS rate was significantly higher in the nPP group compared with the TP group (*P* = 0.001 < 0.017, HR 0.525, 95% CI 0.348–0.791). There were significant differences in ORR among the three groups (71.9% vs. 85.4% vs. 92.2%, *P* = 0.044), and multiple comparisons suggested that the ORR in the nPP group was significantly higher than that in the DDP group (92.2% vs. 71.9%, *P* = 0.013 < 0.017). However, there was no significant difference in ORR between the TP group and the DDP group (85.4% vs. 71.9%, *P* = 0.157); increasing the sample size may be able to observe a statistical difference between the two groups. The cisplatin-alone group in our study seemed to have done much worse than what was reported in the EMBRACE I trial [25], which may be related to the higher clinical stage of the patients in the groups we enrolled. Increasing the number of patients could further refine our study. The incidence of AEs was higher in the TP and nPP groups than in the DDP group. However, the differences were not statistically significant (*P* > 0.05), indicating that the safety of combining paclitaxel or nab-paclitaxel with single-agent cisplatin was tolerable.

A phase II clinical study published by the GOG in 2012 investigated the efficacy and safety of nab-paclitaxel monotherapy in patients with advanced and recurrent cervical cancer. It showed that nab-paclitaxel has considerable activity and moderate toxicity in treating resistant, metastatic, and recurrent cervical cancer [11]. Li et al. [26] employed a combination of nab-paclitaxel and nedaplatin for patients with advanced and recurrent cervical cancer. Their findings showed an ORR of 50.0%, an OS of 16.6 months, a PFS of 9.1 months, and a Grade 3 incidence of thrombocytopenia and anemia at 7.4% and 18.5%, respectively. And no cases of hypersensitivity reactions were reported, suggesting that nab-paclitaxel presents encouraging efficacy and acceptable toxicity profiles. Currently, nab-paclitaxel is approved as a second-line treatment option for patients with recurrent/metastatic cervical cancer. Yu et al. [27] investigated the effect of neoadjuvant chemotherapy consisting of nab-paclitaxel and platinum (NACT-nPP) in patients with LACC. It showed that 72 (92.3%) patients in the NACT-nPP group and 96 (82.1%) patients in the control group achieved CR (*P* = 0.042). Grade 3 or higher acute hematologic AEs were manageable in the NACT-nPP group (46.2%, 36/78), demonstrating the efficacy and safety of nab-paclitaxel neoadjuvant therapy combined with CCRT for LACC. However, despite these findings, there is still debate about whether neoadjuvant therapy confers tangible benefits to LACC patients and currently, most LACC patients continue to receive primary treatment through CCRT [28]. 

This study compared the efficacy and safety of three chemotherapy regimens of single-agent cisplatin, paclitaxel plus cisplatin, and nab-paclitaxel plus cisplatin combined with radiotherapy in the treatment of LACC. The results showed that the two cisplatin-based double-agent chemotherapy regimens were associated with improved outcomes than the single-agent cisplatin regimen, and the AEs were tolerable. Compared with traditional paclitaxel, albumin-bound paclitaxel was associated with improved outcomes in OS, PFS, and ORR of LACC, along with improved treatment compliance of patients. Nonetheless, it’s crucial to acknowledge that this study was a single-center retrospective study, the retrospective nature could introduce potential selection biases. Additionally, the sample size was relatively small, the follow-up period was comparatively short, and at data cutoff, outcomes for many patients remained unknown. Consequently, further validation through larger-scale, prospective studies is required to substantiate these findings.

## Conclusions

For LACC, nPP regimen concurrent with radiotherapy appears to offer superior benefits compared to TP regimen and DPP regimen and adverse reactions are tolerable. The findings from this study can provide valuable reference for the clinical treatment of LACC.

## Data Availability

The datasets generated and/or analysed during the current study are not publicly available due to the experiment is still in progress but are available from the corresponding author on reasonable request.

## Abbreviations

LACC: Locally advanced cervical cancer
DDP: Single-agent cisplatin
TP: Paclitaxel combined with cisplatin
cc: Nanoparticle albumin-bound (nab-) paclitaxel combined with cisplatin
OS: Overall survival
PFS: Progression-free survival
ORR: Objective response rate
AEs: Adverse events
CCRT: Concurrent chemoradiotherapy
ICBT: Intracavitary brachytherapy
MRI: Magnetic resonance imaging
PET-CT: Positron emission computed tomography
CT: Computed tomography
RT: Radiotherapy
EBRT: External beam radiation therapy
RTOG: American Radiation Therapy Oncology Collaborative Group
GTV: Gross target volume
CTV: Clinical target volume
IMRT: Intensity-modulated radiation therapy
EQD2: Equivalent dose of 2 Gy
OAR: Organs at risk
RECIST: Response Evaluation Criteria in Solid Tumors
CR: Complete response
PR: Partial response
SD: Stable disease
PD: Progressive disease
ANOVA: One-way analysis of variance
NCCN: National Comprehensive Cancer Network
GOG: Gynecologic Oncology Group
RTOG: Radiation Therapy Oncology Group
SWOG: Southwest Oncology Group
DFS: Disease-free survival

## References

[1] Sung H (2021). Global Cancer statistics 2020: GLOBOCAN estimates of incidence and Mortality Worldwide for 36 cancers in 185 countries. Cancer J Clin.

[2] Cohen PA, Jhingran A, Oaknin A, Denny L (2019). Cervical cancer. Lancet (London England).

[3] Gennigens C, De Cuypere M, Hermesse J, Kridelka F, Jerusalem G (2021). Optimal treatment in locally advanced cervical cancer. Expert Rev Anticancer Ther.

[4] Naga Ch P, Gurram L, Chopra S, Mahantshetty U (2018). The management of locally advanced cervical cancer. Curr Opin Oncol.

[5] Green JA (2001). Survival and recurrence after concomitant chemotherapy and radiotherapy for cancer of the uterine cervix: a systematic review and meta-analysis. Lancet (London England).

[6] da Costa SCS (2019). Neoadjuvant Chemotherapy with Cisplatin and Gemcitabine followed by Chemoradiation Versus Chemoradiation for locally Advanced Cervical Cancer: a randomized phase II trial. J Clin Oncology: Official J Am Soc Clin Oncol.

[7] McGuire WP, Blessing JA, Moore D, Lentz SS, Photopulos G (1996). Paclitaxel has moderate activity in squamous cervix cancer. A Gynecologic Oncology Group study. J Clin Oncology: Official J Am Soc Clin Oncol.

[8] Della Corte L (2020). Advances in paclitaxel combinations for treating cervical cancer. Expert Opin Pharmacother.

[9] Minion LE, Tewari KS (2018). Cervical cancer - state of the science: from angiogenesis blockade to checkpoint inhibition. Gynecol Oncol.

[10] 10 Kundranda MN, Niu J (2015). Albumin-bound paclitaxel in solid tumors: clinical development and future directions. Drug Des Devel Ther.

[11] Alberts DS (2012). Phase II trial of nab-paclitaxel in the treatment of recurrent or persistent advanced cervix cancer: a gynecologic oncology group study. Gynecol Oncol.

[12] Hsu HC, Li X, Curtin JP, Goldberg JD, Schiff PB (2015). Surveillance epidemiology and end results analysis demonstrates improvement in overall survival for cervical cancer patients treated in the era of concurrent chemoradiotherapy. Front Oncol.

[13] Nagy, V.et al. Radiotherapy versus concurrent 5-day cisplatin and radiotherapy in locally advanced cervical carcinoma. Long-term results of a phase III randomized trial. *Strahlentherapie und Onkologie: Organ der Deutschen Rontgengesellschaft … et al]* 185, 177–183, doi:10.1007/s00066-009-1893-z (2009). 10.1007/s00066-009-1893-z 19330295

[14] Monk BJ (2009). Phase III trial of four cisplatin-containing doublet combinations in stage IVB, recurrent, or persistent cervical carcinoma: a Gynecologic Oncology Group study. J Clin Oncology: Official J Am Soc Clin Oncol.

[15] 15 Schefter T (2014). RTOG 0417: efficacy of bevacizumab in combination with definitive radiation therapy and cisplatin chemotherapy in untreated patients with locally advanced cervical carcinoma. Int J Radiat Oncol Biol Phys.

[16] Tewari KS (2014). Improved survival with bevacizumab in advanced cervical cancer. N Engl J Med.

[17] Colombo N (2021). Pembrolizumab for Persistent, recurrent, or metastatic cervical Cancer. N Engl J Med.

[18] Naumann RW (2019). Safety and Efficacy of Nivolumab Monotherapy in recurrent or metastatic cervical, vaginal, or Vulvar Carcinoma: results from the phase I/II CheckMate 358 trial. J Clin Oncology: Official J Am Soc Clin Oncol.

[19] Mayadev J (2020). Efficacy and safety of concurrent and adjuvant durvalumab with chemoradiotherapy versus chemoradiotherapy alone in women with locally advanced cervical cancer: a phase III, randomized, double-blind, multicenter study. Int J Gynecol cancer: Official J Int Gynecol Cancer Soc.

[20] Lorusso D (2024). Pembrolizumab or placebo with chemoradiotherapy followed by pembrolizumab or placebo for newly diagnosed, high-risk, locally advanced cervical cancer (ENGOT-cx11/GOG-3047/KEYNOTE-A18): a randomised, double-blind, phase 3 clinical trial. Lancet (London England).

[21] Petrelli F, De Stefani A, Raspagliesi F, Lorusso D, Barni S (2014). Radiotherapy with concurrent cisplatin-based doublet or weekly cisplatin for cervical cancer: a systematic review and meta-analysis. Gynecol Oncol.

[22] Ma S (2019). Platinum single-agent vs. platinum-based Doublet agent concurrent chemoradiotherapy for locally advanced cervical cancer: a meta-analysis of randomized controlled trials. Gynecol Oncol.

[23] Marupudi NI (2007). Paclitaxel: a review of adverse toxicities and novel delivery strategies. Exp Opin Drug Saf.

[24] Yardley DA (2013). Nab-Paclitaxel mechanisms of action and delivery. J Controlled Release: Official J Controlled Release Soc.

[25] Pötter R (2021). MRI-guided adaptive brachytherapy in locally advanced cervical cancer (EMBRACE-I): a multicentre prospective cohort study. Lancet Oncol.

[26] Li Y (2017). A phase 2 study of nanoparticle albumin-bound paclitaxel plus nedaplatin for patients with advanced, recurrent, or metastatic cervical carcinoma. Cancer.

[27] Yu XL, Wu MF, Ding L, Yang J, Bai SM (2021). Enhanced efficacy of Neoadjuvant Chemotherapy with Nab-Paclitaxel and Platinum for locally Advanced Cervical Cancer. Cancer Manag Res.

[28] Lapresa M, Parma G, Portuesi R, Colombo N (2015). Neoadjuvant chemotherapy in cervical cancer: an update. Expert Rev Anticancer Ther.

